# Hospital quality reports in Germany: patient and physician opinion of the reported quality indicators

**DOI:** 10.1186/1472-6963-7-157

**Published:** 2007-09-28

**Authors:** Max Geraedts, David Schwartze, Tanja Molzahn

**Affiliations:** 1 Public Health Programme, University Hospital of the Heinrich-Heine-University, Moorenstraße 5, 40225 Düsseldorf, Germany

## Abstract

**Background:**

Starting in 2005, Germany's health law required hospital quality reports to be published every two years by all acute care hospitals. The reports were intended to help patients and physicians make informed choices of hospitals. However, while establishing the quality indicators that form the content of the reports, the information needs of the target groups were not explicitly taken into account. Therefore, the aim of our study was to determine patient and physician opinion of the relevance of the reported quality indicators for choosing or referring to a hospital.

**Methods:**

Convenience samples of 50 patients and 50 physicians were asked to rate the understandability (patients), suitability (physicians) and relevance (both groups) of a set of 29 quality indicators. The set was drawn from the reports (24 indicators) and supplemented by five indicators commonly used in hospital quality reports. We analysed the differences in patient and physician ratings of relevance of all indicators by applying descriptive statistics, t-tests and Wilcoxon tests.

**Results:**

Only three indicators were considered not understandable by the interviewed patients and unsuitable by the interviewed physicians. The patients rated 19 indicators as highly or very relevant, whereas the physicians chose 15 indicators. The most relevant indicator for the patients was "qualification of doctors", and for the physicians "volume of specified surgical procedures". Patient and physician rankings of individual indicators differed for 25 indicators. However, three groups of indicators could be differentiated, in which the relevance ratings of patients and physicians differed only within the groups. Four of the five indicators that were added to the existing set of reported indicators ranked in the first or second group ("kindness of staff", "patient satisfaction", "recommendation", and "distance to place of living").

**Conclusion:**

Most of the content of Germany's hospital quality reports seems to be useful for patients and physicians and influence their choice of hospitals. However, the target groups revealed that approximately one third of the indicators (mostly hospital structural characteristics), were not useful and hence could have been omitted from the reports. To enhance the usefulness of the reports, indicators on patient experiences should be added.

## Background

By law, all German hospitals are required to publish a structured quality report every two years. The first series of reports of 1,983 acute care hospitals was published in PDF-format on the internet in September of 2005 [1]. The aim of these quality reports was to allow insurees/patients and referring physicians to make an informed choice of hospitals [2].

Publishing these reports on the quality of hospital care is comparable to efforts in many countries of the world [e.g. [3-6]]. However, the process of choosing the right quality indicators for the report did not follow state of the art methodology [7-12], as reflected in the set of indicators that was ultimately chosen. A group of representatives of the self-administration of the German health care system – representing the statutory social health insurance funds and the German hospital association – established the content of the reports without explicitly taking into account international experiences and the information needs of patients and referring physicians [Daniela Riese, association of substitute health insurance funds for employees, personal communication].

Therefore, the aim of our study was to examine whether the quality reports and their respective quality indicators could nevertheless be useful for informed decision making by determining the relevance attributed to the reported hospital quality indicators by patients and referring physicians.

## Methods

### Quality indicators

The German quality reports are divided into two parts:

- A structured and comparable part with information on the scope and volume of services provided by each hospital

- An unstructured and virtually comparable part with information on hospital quality management systems.

Since these quality reports do not contain quality indicators as exactly defined parameters [13], we extracted all items from the first part of the reports that may be relevant for informed decision making while choosing a hospital (see list of indicators in the table). To study the usefulness of the structured quality reports as they were published in 2005, i.e. as PDF-files without glossary, we used the exact wording of the reports and explained the meaning of the different indicators to the interviewed patients and physicians in more detail only on request and only after receiving their answers to our questions.

The reports missed many indicators that are commonly used in quality reports and we did not want to excessively strain our interviewees. We therefore added only five internationally acknowledged indicators to the former list of 24 indicators: "patient satisfaction", "kindness of staff", "distance to place of living", "recommendation by relatives or physicians", and "hospital regularly visited or referred to". We thus ended up with 29 indicators to be examined in our study.

### Patients, physicians and settings

Since surveys that do not collect personal data do not require approval by the ethics committee of the University of Duesseldorf, we only asked the interviewees for their consent to participate in the study. On four consecutive days, a medical student interviewer (DS) visited the waiting rooms of four general practitioners' surgeries in the cities of Cologne, Duesseldorf, Wuppertal, and Duisburg, which are located in the Rhine-Ruhr area of Germany. There, he recruited 50 patients who agreed to take part in an interview on choosing hospital care. The sample was therefore drawn as a convenience sample of consecutive patients of the respective private practices.

Table 1 presents the patient group's age and gender structure, as well as the proportion of the proportion of statutorily insured study patients compared to this proportion in average GP patients in Germany [Zentralinstitut für die kassenaerztliche Versorgung, Berlin: Patient-Physician-Panel (ADT-Panel), I/2007, personal communication]. In the study patient group, women and patients holding private insurance were overrepresented. Concerning experience with in-hospital care in Germany, data for the average group of a GP's patients do not exist. In our sample, two patients did not yet experience a hospital stay, the remaining 48 experienced an average of 4.7 periods of hospital care (range of 1–30 episodes of in-hospital care).

**Table 1:** Socio-demographic data of study patients compared to average GP patients in Germany

	Study patients	Average GP patients
Mean age (range)	53 years (18–81)	50 years (0–>100)
% women	68%	57%
% statutorily insured	68%	90%

DS performed the 50 structured face-to-face interviews that took an average of 25 minutes in a separate room of the doctor's office. The patients were asked to imagine suffering from an inguinal hernia. In order to choose a hospital for performing the required surgery, they were handed a fictitious "regional hospital quality information package". This package was comprised of different comparative graphs on the quality of care at ten hospitals along with the list of indicators that were used to compare the hospitals. The patients were then asked to rate a) the understandability of the indicators (understandable/not understandable), and b) the relevance of the 29 indicators for making an informed choice of which hospital to go to. The rating scale ranged from 1–6 and was based on the grading system in German schools:

- One (1) = highly relevant

- Two (2) = very relevant

- Three (3) = somewhat relevant

- Four (4) = somewhat irrelevant

- Five (5) = very irrelevant

- Six (6) = completely irrelevant

Another medical student interviewer (TM) visited 50 general practitioners (GPs) who agreed to take part in the study. All practitioners worked in the cities of Hagen and Bochum and their surrounding regions in North Rhine-Westphalia. The convenience sample was drawn by informing all practitioners (N = 164) of those two regions about the study in writing and choosing the first 50 that agreed to participate. Their mean age was 50 years and 10 were women. Only 13 worked in group practices; the remaining 37 in single private practices.

The GPs were asked to imagine a patient needing an inguinal hernia operation. In order to make an informed referral decision, the physicians received a fictitious information package from the region's social health insurance funds. This package was comprised of information about the quality of care in regional hospitals. The physicians were asked to rate a) the suitability (yes/no) and, b) the relevance of the 29 indicators for making an informed referral decision (rating scale from 1 to 6 as explained above).

### Analysis

We compared the number of patients who rated the different indicators as understandable and the number of physicians who rated the different indicators as suitable. The mean ratings and standard deviations of every indicator were examined with respect to their relevance rating. We analysed differences between the patient and physician ratings of relevance of the individual indicators by applying t-tests. Within the groups of patients and physicians, we analysed the ratings by performing Wilcoxon tests, comparing the ratings of those neighbouring indicators that differed in their median ratings between grades two and three.

## Results

Table 2 (see additional file 1) shows each of the 29 indicators with the ratings of patients and physicians in relation to their relevance, understandability and suitability. The indicators are ranked according to the mean relevance ratings of the patient group, showing the highest ranking indicator ("qualification of doctors") at the top of the list. In addition, the table shows the relevance ranking by the physician group.

Considering understandability, most indicators (22 out of 29) were understandable for more than 40 interviewed patients (80%). However, only five indicators were understood by the entire group of patients. In the physician group, only one indicator was suitable for all of the interviewed doctors and only 11 out of 29 indicators were suitable for more than 80% of them. Four indicators were judged as not understandable by more than half of the patients compared to seven indicators deemed not suitable in the group of physicians.

Considering the patient relevance ratings for each of the indicators, 19 indicators were rated highly or very relevant (median grades one or two). The remaining 10 received grades of three to six, meaning somewhat relevant to completely irrelevant. Only one indicator received a median grade of four ("hospital owner"). However, 16 out of 29 indicators were graded completely irrelevant by at least one of the interviewed patients.

The majority of physicians rated fifteen indicators as highly or very relevant and fourteen as somewhat relevant to completely irrelevant. Five indicators received a median grade of four or five. 24 out of 29 indicators were graded as completely irrelevant by at least one physician.

Within the groups of patients and physicians, the indicator ratings differed significantly for those indicators that were rated grade three (somewhat relevant) ("professional training for doctors" in both groups) compared to the indicators with a median rating of grade two (very relevant) or better (in the patients, the indicator ranked on position 18 "distance to place of living", in the physicians, the indicator ranked on position 12 and better "specialist outpatient department").

The comparison of patient and physician relevance rankings shows no difference for four indicators, whereas the rankings of all other indicators differed between one and seven positions. However, the analysis indicates three groups of indicators that may be differentiated:

- Indicators ranked 1^st ^to 10^th ^position ("qualification of doctors" to "range of therapeutic facilities"),

- Indicators ranked 11^th ^to 19^th ^position ("recommendation" to "professional training for doctors")

- Indicators ranked 20^th ^to 29^th ^position ("hospital operates an outpatient department" to "hospital owner").

The relevance rankings of patients and physicians differed only within these groups and did not overlap with the other groups. Within the first group of ten indicators, only one showed a significant difference between the patient and physician ratings of relevance. In both the second and third group of indicators, four differed significantly.

The first ten indicators were judged most relevant by both groups. However, while the patients ranked the indicators "qualification of doctors", "kindness of staff" and "patient satisfaction" in the 1^st ^to 3^rd ^positions, these indicators received the 6^th^, 7^th ^and 5^th ^rank respectively, by the group of physicians. On the other hand, the physicians ranked the indicators "volume of specified surgical procedures", "24-hours-availability of technical equipment", and "range of therapeutic facilities" 1^st ^to 3^rd^, whereas the patients ranked these indicators on the 7^th^, 6^th^, and 10^th ^position, respectively.

Five indicators were added to the list published in the official German hospital quality reports. Two of them ranked in the first group ("kindness of staff" and "patient satisfaction") received 2^nd ^and 3^rd ^positions from the patient group and 7^th ^and 5^th ^position from the physicians. Two in the second group ("recommendation" and "distance to place of living") received 11^th ^and 18^th ^positions from the patient group and 14^th ^and 13^th ^position from the physicians. The last in the third group ("hospital regularly visited or referred to") received the 25^th ^position from the patient group and the 27^th ^position from the physicians.

## Discussion

Most but not all indicators published recently as part of the first series of obligatory federal hospital quality reports in Germany were judged by patients and referring physicians as useful in choosing a hospital. At least 10 out of 24 indicators could be omitted from the quality reports because both target groups (patients and physicians) rated these indicators as less relevant for choosing a hospital. In addition, three of these indicators were not understandable or suitable to more than half of both target groups, further justifying their omission. However, at least three indicators ("kindness of staff", "patient satisfaction", and "recommendation") should be added to the reports in order to enhance their usefulness. They received high relevance and at the same time high understandability or suitability rankings.

### Understandability and suitability of the indicators

The interviewed patients apparently understood almost all indicators included in the hospital quality reports. Only four indicators were judged "understandable" by less than half of the patients. The physicians' judgements of suitability of the listed indicators as an information tool for their referral decision were more diverse. There was only one indicator deemed suitable to all physicians ("volume of specified surgical procedures") and only eleven indicators were suitable to more than 80% of the physicians.

### Relevance of the indicators

The patients judged the indicators more positively than the physicians by rating a greater number of indicators as highly or very relevant for choosing a hospital. Only one indicator received a median grade of four by the patients. The number of patients treated as well as some structural characteristics of hospitals (e.g. "certified to treat insured accidents") appeared to be of less relevance to patients. By way of contrast, patients considered the number of procedures performed and the range and availability of specialty services as very relevant. Especially the qualification of doctors and nurses and indicators assessing patient satisfaction seemed to be relevant for choosing a hospital. The kindness of the staff and the reputation of a hospital were also highly relevant to patients.

The indicators concerning patient experiences played an important role for physicians as well. However, the top four positions in the physician ranking of relevance were held by indicators focussing on the number of procedures and range and availability of services. Those indicators judged less relevant by the patients were judged even less relevant by the physicians.

However, based on the comparison of the rankings depicted in the table, the patient and physician rankings did not differ entirely. There were three groups of indicators within which the positions of the individual indicators differed marginally between the patients and physicians but did not overlap with the other groups (positions 1 to 10, 11 to 19, and 20 to 29). Especially the indicators ranked position 1 to 10 in both groups seemed to be highly useful for hospital quality reports. Within this group, only one indicator differed significantly between patients and physicians. Based on these results, the quality reports could be condensed by omitting at least the third group of indicators (positions 20–29) judged more or less irrelevant by both groups. At the same time, the quality reports should be expanded by incorporating those indicators of patient experiences that were rated highly or very relevant by both patients and physicians.

### Generalisation of the results

Some methodological issues question the generalisation of the results. First of all, the small group of interviewees (convenience samples of 50 patients and 50 physicians) might have caused a selection bias. In the group of patients, women and privately insured individuals were overrepresented. Private insurance is usually correlated to social class in Germany, thereby creating an overrepresentation of higher social class patients in that group. This may be an explanation for the small differences between the patient and physician ratings of the indicator relevance and for the high ratings of understandability in the group of patients. There might have been much higher differences in the indicator ratings if a more representative sample of the German population had been questioned.

However, the results of our small scale study have recently been supported by a survey of a representative sample of the German adult population. Out of 33 indicators that were rated according to their relevance for choosing a hospital, those indicators that were rated highly or very relevant in our study were rated almost the same way by the representative population sample [14].

In addition, the overrepresentation of privately insured individuals reflects the actual users of the hospital quality reports. These reports were exclusively published via the internet and higher social class individuals generally tend to show higher internet access and use in Germany.

In regard to the physicians, the group of interviewees were well represented by general practitioners (GPs) in Germany. German GPs are required by law to counsel patients and support them in choosing a hospital for treatment. This provides justification for the selection of GPs and the exclusion of specialized physicians for the study. However, interviewing the first 50 GPs who agreed to participate in the study might have caused a bias, since these physicians might have judged the suitability and relevance of the indicators more positively than a non-convenience sample of GPs.

Moreover, the lack of detailed explanations of the indicators provided to the interviewees before collecting their ratings could have led to misunderstandings. This could have caused false results. As an example, there were significant differences between the ratings of relevance for the indicators "realization of minimum volume standards" (patients: 23^rd ^position, physicians: 21^st ^position) and "volume of specified surgical procedures" (patients: 7^th ^position, physicians: 1^st ^position). However, the meaning of both indicators overlap to a great extent: the first indicator shows whether a hospital fulfils the volume requirements set by law for five specific surgical procedures, the second indicator stands for the volume of surgical procedures in general. Since the indicator "realization of minimum volume standards" has been judged not understandable by two thirds of the patients, this large difference might well be explained. Because most other indicators were considered understandable, the problem of misunderstandings might be negligible. And since the interview setting – not commenting the indicators before the interviewees rated understandability (patients) or suitability (physicians) and relevance (both groups) – corresponds to the situation in which the patient or physician performs an internet information search of the quality reports, the results of our study may be especially valid. Even when using the internet, patients and physicians might not be able to find additional explanatory information.

Finally, the study results might be of limited value because the indicators of the German hospital quality reports reflect unique features of the German health care system and only marginally compare to indicators used in other countries. Indeed, most of the indicators refer to structural characteristics of hospitals that are specific to Germany. Maintaining an outpatient department is usually only permitted in bigger hospitals, for example. Only those indicators that have been added reflect some qualitative features of hospitals that are commonly measured in performance assessment. In addition, the methodology of setting up the German hospital quality report did not follow state of the art approaches [7-12]. There might be no protocol that uses identical indicators.

Nevertheless, some lessons remain to be learned if one looks at the meaning of the indicators rather than at the individual indicator itself. Patients and physicians as target groups of hospital quality reports preferred indicators of range, volume and availability of services in hospitals, and of staff qualification and patient experiences over indicators that only state numbers of cases, structures or equipment. Since stakeholders around the world are struggling to issue comparable information on the quality of hospitals, it might be worthwhile not only to follow internationally acknowledged methodology but also to consider the views of patients and physicians as principal target groups of the reports.

## Conclusion

Considering the understandability and relevance ratings of the indicators that form the basis of the German hospital quality reports, most of the report content seems to be useful to patients and physicians in their choice of hospitals. Participation of the target groups in the design of the quality reports could have revealed that about one third of the indicators were not useful and hence could have been omitted. In addition, this study shows that at least some qualitative indicators on patient experiences should have been added to the reports in order to enhance their usefulness for both patients and physicians.

## Competing interests

The author(s) declare that they have no competing interests.

## Authors' contributions

MG conceived and designed the study, supervised data analysis and drafted the manuscript. DS collected the data, performed data analysis and revised the manuscript critically for important intellectual content. TM collected the data, performed data analysis and revised the manuscript critically for important intellectual content. All authors read and approved the final manuscript.

## Pre-publication history

The pre-publication history for this paper can be accessed here:



## Supplementary Material

Additional file 1 Table 2: Patient and physician opinion of quality indicators for choosing hospitals. Patient and physician ratings of the relevance, understandability and suitability of hospital quality indicators to choose or refer to a hospital (N = 50 patients, 50 physicians); indicators are ranked according to the mean ratings of relevance by patients. Click here for file

## References

[B1] Annahmestelle für die strukturierten Qualitaetsberichte der Krankenhaeuser Qualitaetsberichte fuer 2004 und 2005. http://www.g-qb.de.

[B2] Federal Joint Committee Vereinbarung gemaess §137 Abs. 1 Satz 3 Nr. 6 SGB V ueber Inhalt und Umfang eines strukturierten Qualitaetsberichts fuer nach §108 SGB V zugelassene Krankenhaeuser. BAnz 22122005.

[B3] Hospital Report Research Collaborative Public reports by year. http://www.hospitalreport.ca/downloads/annual.html.

[B4] Joint Commission on Accreditation of Healthcare Organizations Qualitycheck. http://www.qualitycheck.org.

[B5] National Health Service Performance ratings 2005. http://ratings2005.healthcarecommission.org.uk/home.asp.

[B6] Marshall MN, Shekelle PG, Davies HTO, Smith PC (2003). Public reporting on quality in the United States and the United Kingdom. Health Affairs.

[B7] McGlynn EA (2003). Selecting common measures of quality and system performance. Med Care.

[B8] Veillard J, Champagne F, Klazinga N, Kazandjian V, Arah OA, Guisset AL (2005). A performance assessment framework for hospitals: the WHO regional office for Europe PATH project. International Journal for Quality in Health Care.

[B9] Galvin RS (1998). Are performance measures relevant?. Health Affairs.

[B10] Marshall MN, Romano PS, Davies HTO (2004). How do we maximize the impact of the public reporting of quality of care?. International Journal for Quality in Health Care.

[B11] Vaiana ME, McGlynn EA (2002). What cognitive science tells us about the design of reports for consumers. Med Care Res Rev.

[B12] Mainz J (2003). Developing evidence-based clinical indicators: a state of the art methods primer. International Journal for Quality in Health Care.

[B13] Geraedts M, Selbmann HK, Ollenschlaeger G (2003). Critical appraisal of clinical performance measures in Germany. International Journal for Quality in Health Care.

[B14] Geraedts M, Böcken J, Braun B, Amhof R, Schnee M (2006). Qualitätsberichte deutscher Krankenhäuser und   Qualitätsvergleiche von Einrichtungen des Gesundheitswesens aus   Versichertensicht.. Gesundheitsmonitor 2006 Gesundheitsversorgung und Gestaltungsoptionen aus der Perspektive von Bevölkerung und Ärzten.

