# Supplementary material for: Hospital quality reports in Germany: patient and physician opinion of the reported quality indicators
Source: BMC Health Serv Res. 2007 Sep 28;7:157. doi: 10.1186/1472-6963-7-157 (PMC2048956; doi:10.1186/1472-6963-7-157)
Supplement: Additional file 1 — Table 2: Patient and physician opinion of quality indicators for choosing hospitals. Patient and physician ratings of the relevance, understandability and suitability of hospital quality indicators to choose or refer to a hospital (N = 50 patients, 50 physicians); indicators are ranked according to the mean ratings of relevance by patients. [file 1472-6963-7-157-S1.pdf]

Table 2: Patient and physician opinion of quality indicators for choosing hospitals

Patient and physician ratings of the relevance[1], understandability and suitability[2] of hospital quality indicators to choose or refer to a hospital (N = 50 patients, 50 physicians); indicators are ranked according to the mean ratings of relevance by patients

**Indicator**	**Patients**			**Physicians**		
	**Rank**	**Relevance** mean – SD - median	**Understand-able** (%)	**Rank**	**Relevance** mean - SD - median	**Suitable** (%)
Qualification of doctors	1	1.47 - 0.77- 1	86	6	1.76 - 1.02 - 2	84
Kindness of staff	2	1.54 - 0.65 - 1	100	7	1.82 - 1.08 – 1.5	92
Patient satisfaction	3	1.60 - 0.70 – 1.5	100	5	1.74 - 1.12 - 1	90
Range of technical equipment	4	1.61 - 0.77 - 1	94	8	2.08 - 1.18 – 2*	84
Qualification of nurses	5	1.76 - 0.86 - 2	92	10	2.14 - 1.26 - 2	76
24-hours-availability of technical equipment	6	1.79 - 0.94 - 2	90	2	1.70 - 0.93 - 1	88
Volume of specified surgical procedures	7	1.83 - 0.84 - 2	98	1	1.58 - 0.79 - 1	100
Specialist departments range of services	8	1.86 - 0.89 - 2	88	4	1.72 - 0.95 - 2	88
Medical services and special offers	9	1.91 - 0.83 - 2	86	9	2.14 - 1.05 - 2	88
Range of therapeutic facilities	10	1.93 - 0.72 - 2	92	3	1.72 - 1.01 – 1.5	86
Recommendation by relatives or physicians	11	1.96 - 0.96 - 2	100	14	2.62 - 1.35 – 2*	72
Specialist outpatient department	12	2.19 - 1.09 - 2	86	11	2.20 - 1.09 – 2	92
Participation in external quality assurance	13	2.23 - 1.22 - 2	64	19	3.18 - 1.56 – 3*	66
Volume of specified out-patient operations	14	2.24 - 1.18 - 2	96	12	2.48 - 1.23 – 2	78
Hospital’s number of specialist departments	15	2.24 - 1.26 - 2	100	17	2.94 - 1.30 – 3*	64
Outpatient treatment facilities	16	2.30- 1.28 - 2	92	15	2.72 - 1.26 – 2	60
Results of external quality assurance	17	2.31 - 1.23 - 2	60	18	3.12 - 1.52 – 3*	60
Distance to place of living	18	2.49 - 1.12 - 2	98	13	2.54 - 1.18 – 2	82
Professional training for doctors	19	2.61 - 1.40 - 3	74	16	2.80 - 1.41 – 3	58
Hospital operates an outpatient department	20	2.75 - 1.29 - 3	48	20	3.26 - 1.59 – 3	58
Participation in Disease Management Programmes	21	2.88 - 1.17 - 2	34	28	4.58 - 1.40 – 5*	24
Academic teaching hospital	22	2.90 - 1.39 - 3	84	23	3.46 - 1.47 – 3	66
Realization of minimum volume standards	23	3.00 - 1.28 - 3	34	21	3.44 - 1.57 – 3	44
Hospital’s total number of inpatient cases	24	3.12 - 1.41 - 3	98	22	3.44 - 1.25 – 3	52
Hospital regularly visited or referred to	25	3.18 - 1.50 - 3	86	27	4.12 - 1.41 – 4*	34
Hospital’s total number of beds	26	3.22 - 1.35 - 3	100	26	3.82 - 1.30 – 4*	40
Hospital’s total number of outpatient cases	27	3.41 - 1.22 - 3	92	25	3.80 - 1.39 – 3.5	36
Certified to treat insured accidents	28	3.60 - 1,27 - 3	40	24	3.70 - 1.43 – 4	46
Hospital owner	29	3.63 - 1.46 - 4	88	29	4.82 - 1.41 – 5*	8

[1]: relevance rating: 1 to 6; 1 = highly relevant; 6 = completely irrelevant; table shows mean, standard deviation (SD) and median

[2]: understandability: % of patients rating the indicator as “understandable”; suitability: % of physicians rating the indicator as “suitable”

*: differences between the patient and physician ratings of relevance significant ($p<.05$, T-test)
